# Evaluation of the use of CRISPR loci for discrimination of *Salmonella enterica* subsp. *enterica* serovar Enteritidis strains recovered in Canada and comparison with other subtyping methods

**DOI:** 10.3934/microbiol.2022022

**Published:** 2022-07-15

**Authors:** Susan Nadin-Davis, Louise Pope, John Devenish, Ray Allain, Dele Ogunremi

**Affiliations:** Ottawa Laboratory Fallowfield, Canadian Food Inspection Agency, Ottawa, ON, Canada

**Keywords:** *Salmonella enterica*, serovar, *S*. Enteritidis, subtyping, phage, PFGE, CRISPR, SNP-PCR

## Abstract

*Salmonella enterica* subsp. *enterica* serovar Enteritidis remains one of the most important foodborne pathogens worldwide. To minimise its public health impact when outbreaks of the disease occur, timely investigation to identify and recall the contaminated food source is necessary. Central to this approach is the need for rapid and accurate identification of the bacterial subtype epidemiologically linked to the outbreak. While traditional methods of *S*. Enteritidis subtyping, such as pulsed field gel electrophoresis (PFGE) and phage typing (PT), have played an important role, the clonal nature of this organism has spurred efforts to improve subtyping resolution and timeliness through molecular based approaches. This study uses a cohort of 92 samples, recovered from a variety of sources, to compare these two traditional methods for *S*. Enteritidis subtyping with recently developed molecular techniques. These latter methods include the characterisation of two clustered regularly interspaced short palindromic repeats (CRISPR) loci, either in isolation or together with sequence analysis of virulence genes such as *fim*H. For comparison, another molecular technique developed in this laboratory involved the scoring of 60 informative single nucleotide polymorphisms (SNPs) distributed throughout the genome. Based on both the number of subtypes identified and Simpson's index of diversity, the CRISPR method was the least discriminatory and not significantly improved with the inclusion of *fim*H gene sequencing. While PT analysis identified the most subtypes, the SNP-PCR process generated the greatest index of diversity value. Combining methods consistently improved the number of subtypes identified, with the SNP/CRISPR typing scheme generating a level of diversity comparable with that of PT/PFGE. While these molecular methods, when combined, may have significant utility in real-world situations, this study suggests that CRISPR analysis alone lacks the discriminatory capability required to support investigations of foodborne disease outbreaks.

## 1. Introduction

Foodborne gastroenteritis is a major public health concern globally, resulting in significant morbidity, mortality and economic losses [1], with certain serovars of *Salmonella enterica* subsp. *enterica* figuring prominently [2] due to their wide dispersal in water sources used in agriculture [3]. In many developed countries, including Canada and the USA, *S*. Enteritidis is responsible for a significant proportion of all laboratory-confirmed cases of human salmonellosis [4]–[6] and has been associated with several large disease outbreaks resulting from contamination of fresh produce and poultry products [1],[7]–[10]. Indeed, poultry meat and egg products have been recognised as a significant exposure risk [8],[11] and have been the subject of major food recalls [12]. National programmes aim to prevent contamination of products at the source [13], but when these fail, timely identification of the food source of the disease-causing bacteria and removal of the product from the food supply system are instrumental in limiting the impact on public health.

A key requirement for the successful implementation of an investigation into foodborne salmonellosis is the accurate identification of the bacterial strain epidemiologically linked with both food contamination and human disease. To this end, several subtyping methods have been developed with the aim of devising cost-effective protocols that are timely and reproducible in a variety of laboratory settings [14]. One of the earliest subtyping schemes was phage typing, in which strains are evaluated for susceptibility to a panel of bacteriophages [15]. However, this method could be undertaken by only a limited number of laboratories due to the need for access to a panel of well characterised phages; furthermore, it has been found to lack robust epidemiological capability [16]. The development of pulsed field gel electrophoresis (PFGE), which characterises whole genomes based on the band patterns produced upon treatment of DNA with selected restriction endonucleases, provided a useful alternative that, until very recently was the gold standard technique, widely applied to *Salmonella* in food outbreak investigations [17],[18]. However, it has limited capability to differentiate *S*. Enteritidis strains. An alternative subtyping technique, multiple-locus variable number tandem repeat analysis (MLVA), which scores the number of short tandem repeats present at several different defined loci throughout the genome, has been developed into a standardised protocol employed for epidemiological investigations of *S*. Enteritidis worldwide, often in conjunction with PFGE [19],[20]. Multi-locus sequence typing (MLST), which involves determination of the nucleotide sequences for a set of housekeeping genes, was proposed as a novel means of discriminating between closely related micro-organisms [21]. Its use as an alternative to serotyping for identification of *S. enterica* strains was proposed [22], but it was insufficiently discriminatory for use in foodborne outbreak investigations.

Since the more widespread availability of whole genome sequencing (WGS), there has been a shift towards replacement of these traditional tools by *in silico* analyses of WGS data to enable both serovar identification [23] and highly sensitive strain subtyping through methods such as core genome MLST [24]. While WGS provides for the most comprehensive genetic characterisation of a strain, the technology remains reasonably expensive per strain, requires considerable bioinformatics, human and material resources, and may take many days to complete. Methods which use WGS data to devise simpler and faster tools for strain subtyping have thus also been developed. These include an SNP-based approach in which nucleotide polymorphisms at several specific locations across the genome are evaluated by a PCR-based assay to provide a dataset used for phylogenetic assessment [25].

Yet another subtyping approach which has gained interest for application to many bacterial pathogens involves sequence analysis of clustered regularly interspaced short palindromic repeats (CRISPR) loci [26]. This sequence element is considered part of an innate immune system utilised by many bacteria, including *Salmonella*, to prevent invasion by mobile genetic elements and phages through the identification and subsequent degradation of foreign DNA [27]. Each CRISPR locus consists of several direct repeat sequences which are interspersed by spacer sequences derived from foreign DNA. A set of CRISPR-associated (*Cas*) genes is responsible for mediating this protective mechanism through processing of non-native DNA into short segments that are integrated as spacer sequences into the CRISPR locus. Following transcription of this locus, one or more cas proteins cleave the RNA at the spacer sequences, to generate small interfering RNA/protein complexes that identify complementary unintegrated DNA sequences and elicit their subsequent cleavage and degradation. This process thereby prevents persistence and propagation of foreign genetic material within the cell [28]. The acquisition or loss of spacer sequences in the CRISPR locus reflects the challenge history of the strain, and thus characterisation of this sequence element would be expected to provide a sensitive subtyping target. Indeed, comparative genomics of *S. enterica* strains, which harbour two CRISPR loci, suggested the importance of CRISPR-mediated immunity in regulating gene acquisition from mobile genetic elements and thereby influencing lineage evolution and diversification [29]. Studies showing that CRISPR polymorphism correlated well with *Salmonella* serotype and MLST designation also suggested the utility of these loci for serovar subtyping [30],[31]. A method designated CRISPR-including multi-virulence-locus sequence typing (CRISPR-MLVST), which includes sequence characterisation of both CRISPR loci and two virulence genes (*fimH* and *sseL*), was described for subtyping of several *Salmonella* serovars, including *S*. Enteritidis [32],[33]. Similar protocols have been employed to subtype other serovars of significant public health importance, including *S*. Typhimurium and *S*. Heidelberg [31],[34]. An Australian study found that the CRISPR and prophage profiles of a collection of *S*. Typhimurium strains correlated with core genome evolution [35]. Subtyping of *S*. Enteritidis isolates obtained in China, by CRISPR analysis only, identified several subtypes recovered from different poultry production areas [36] and suggested the pathogen's transmission from swine and poultry to humans [37]. In contrast to these reports, other studies have concluded that CRISPR loci of many bacteria, including those of *Salmonella*, exhibit quite limited sequence variation and may no longer be actively accumulating spacer sequences, thus making these loci relatively poor epidemiological markers [38],[39].

To explore this issue further, this study used a collection of *S*. Enteritidis strains, recovered from a variety of environmental and food sources across Canada, to determine the value of strain subtyping by both CRISPR locus and *fim*H gene characterisation compared to the traditional methods of PFGE, PT and an SNP-PCR approach previously developed by our group [25]. CRISPR analysis alone was found to be the least sensitive of all the subtyping methods analysed, though when combined with other methods such as SNP-based typing, high levels of strain discrimination could be achieved.

## 2. Methods

### 2.1. Source of strains and their isolation

This study included a total of 89 *S*. Enteritidis field strains and three reference strains, as detailed in Table 1. In addition, the well characterised *S*. Enteritidis reference strain P125109 (GenBank accession NC_011294) was included in the CRISPR analysis to confirm that our analyses yielded results in complete concordance with previously reported CRISPR sequences. Many of these strains were recovered from environmental swabs taken in poultry production facilities as part of an animal health programme to monitor these operations for *Salmonella* contamination. Culturing of these swabs and identification of *S*. Enteritidis were undertaken as previously described [40]. A few others were isolated from food samples or food processing facilities using standard *Salmonella* isolation procedures, comprising both pre-enrichment and enrichment steps and plating on selective agar plates [41]. Confirmation of serotype and phage typing were undertaken at the *Salmonella* reference laboratory of the Public Health Agency of Canada (PHAC) in Guelph, Ontario.

### 2.2. DNA extraction, PCR and CRISPR sequencing

Pure isolates of *S*. Enteritidis were grown on tryptic soy agar plates and incubated at 36 °C for 18–22 hrs. Cells were scraped off the agar surface and resuspended in phosphate buffered saline prior to total DNA extraction using a Wizard genomic DNA isolation kit as per the supplier's directions (Promega, Madison, Wisconsin). Purified DNA solutions were quantified spectroscopically using a Nanovue instrument (GE Biosciences) and stored at −20 °C.

The two CRISPR loci, CRISPR-1 and CRISPR-2, were amplified separately by PCR using the primer pairs described by Liu and colleagues [33], as detailed in Table 2. Each 50 µL PCR reaction contained 1X PCR buffer, 1.5 mM MgCl_2_, 0.2 mM dNTP, 2.5 U *Taq* DNA polymerase (all supplied by Invitrogen Life Technologies), 1 ng of DNA template and 0.5 µM forward and reverse primers (synthesised by Integrated DNA Technologies, Coralville, Iowa). Amplifications were performed on a GeneAmp 9700 thermocycler (Applied Biosystems, ThermoFisher) using the following cycling conditions: an initial denaturation at 94 °C for 2 min followed by 30 cycles of 94 °C for 1 min, 52 °C for 1 min, 72 °C for 1 min 15 sec and a final extension at 72 °C for 5 min. Product generation was verified by standard gel electrophoresis, and amplicons were purified using a Wizard PCR purification kit (Promega, Madison, Wisconsin) as per the manufacturer's directions.

**Table 1.**
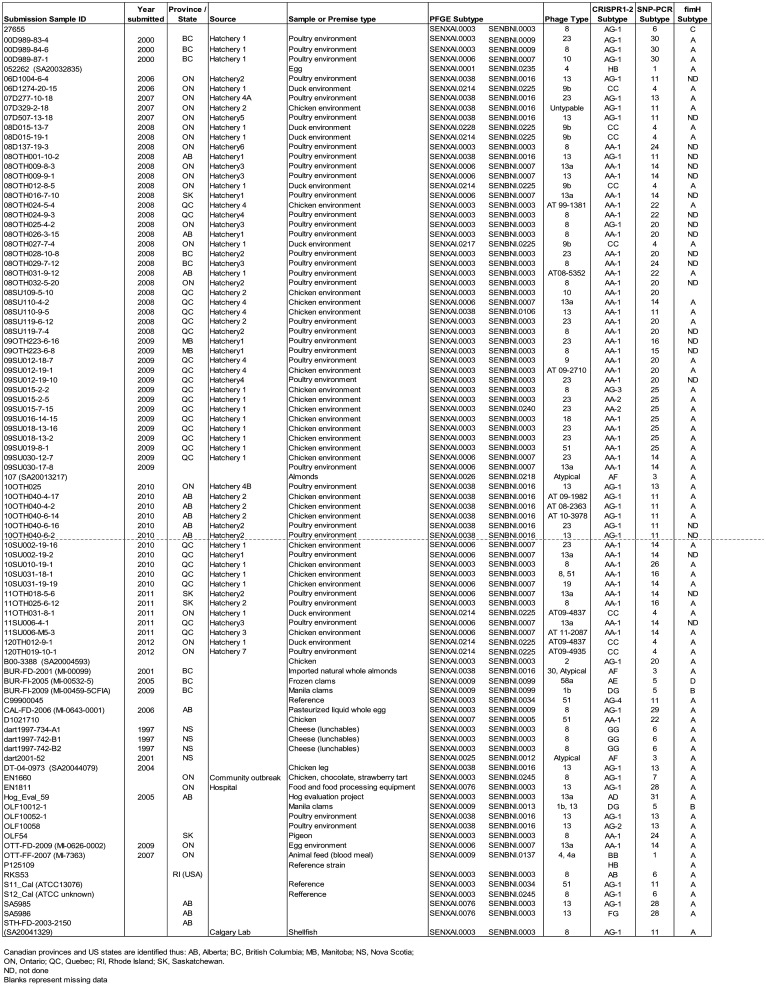
Listing of all *Salmonella* Enteritidis samples and their subtyping classifications.

**Table 2.**
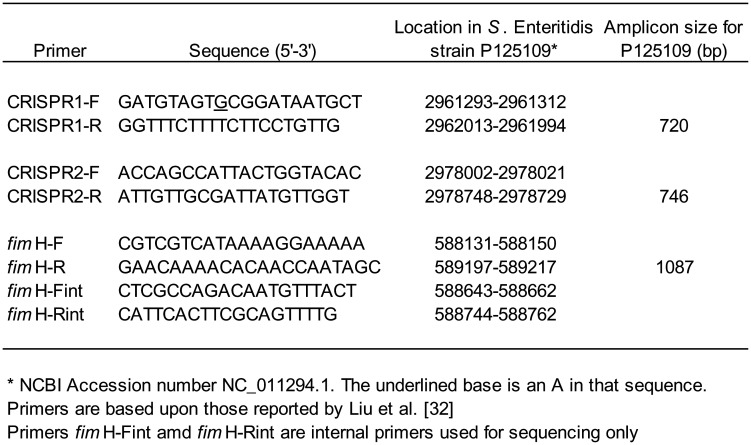
Primers employed for amplification and sequencing of CRISPR and *fimH* loci.

PCR products were sequenced in both directions using the PCR primers with a BDTv3.1 cycle sequencing kit (Applied Biosystems) and a 9700 thermocycler as per kit instructions. Sequencing products were purified using a BDXterminator purification kit (Applied Biosystems) and analysed on a 3500xl genetic analyser (Applied Biosystems). Forward and reverse sequence reads were assembled into a consensus sequence using the Lasergene v.11 software package (DNASTAR Inc., Madison, Wisconsin).

### 2.3. CRISPR analysis

Sequence alignments for each of the two loci were performed using MEGA X [42]. CRISPR loci were analysed using the CRISPRFinder tool [43] available at https://crisprcas.i2bc.paris-saclay.fr to identify the conserved direct repeat (DR) motif and the intervening spacer sequences, each of which was identified numerically. For each strain, the complete CRISPR locus was assigned a single letter according to the spacer sequences it retained, and the combination of scores for both CRISPR loci yielded the final double letter strain type. For some samples, a number was also included to identify minor sequence variations to type. The sequence data from both loci were assembled into a concatenated alignment for generation of a phylogenetic tree using BioNumerics v. 6.01 software (Applied Maths, Sint-Martens-Latem, Belgium).

### 2.4. fimH amplification and sequencing

To explore if additional subtype information could be realised through sequence analysis of the virulence-associated *fim*H gene, this locus was amplified from genomic DNA prepared from 70 samples. The 1087 bp PCR product was generated using the *fim*H-F and *fim*H-R primer pair, which straddles the 1008 bp ORF [32] (Table 2), and procedures similar to those used for the CRISPR loci. Amplicons were sequenced from both DNA strands using *fim*H PCR and internal primers (*fim*H-Fint and *fim*H-Rint), and the reads were assembled using DNASTAR Lasergene v.11 software. An alignment of all assembled sequences was generated in MEGA X to identify all *fim*H gene SNPs using the P125109 strain sequence as the reference, which was designated as allele A. All other alleles thus identified were assigned a single letter designation from B to D (Table 3). Table 1 summarises the *fim*H types of all 70 samples thus analysed.

**Table 3.** Summary of SNPs identified in the *fim*H gene.

*fim*H allele	Sample(s)	Location and nature of SNP in *fim*H ORF	CRISPR subtype
B	BUR-FI-2009 OLF10012-1	A49 to G49 G112 to A112 C259 to T259 C292 to T292 A730 to G730 C770 to T770 T794 to G794	DG
C	27655	T466 to C466	AG-1
D	BUR-FI-2005	T878 to C878	AE

Differences from the sequence of the reference strain P125109 (positions 588156 to 589163), which was assigned as allele A, are indicated. The CRISPR subtype for each of the four samples is also indicated.

### 2.5. PFGE

PFGE was performed using a standard method [44] modified as described [40]. Samples were electrophoresed for 20 hrs on the CHEF Mapper (Bio-Rad Laboratories, Mississauga, ON), and the data were analysed using the BioNumerics v6.01 software. PFGE patterns were assigned by PulseNet Canada (National Microbiology Laboratory, Winnipeg, Canada).

### 2.6. SNP-PCR analysis

For each strain, a set of 60 base positions scattered throughout the genome was determined as described [25] and used to generate a fasta file. An alignment of these fasta files was used to construct a phylogenetic tree using the unweighted pair group method with arithmetic mean (UPGMA), with the MEGA X software employing 1000 bootstrap replicates.

### 2.7. Statistical analysis

The discriminatory capabilities of individual and combined methods were assessed using Simpson's index of diversity [45], calculated using the formula

$$Ds=1-\frac{\sum n_{i}\left(n_{i}-1\right)}{N\left(N-1\right)}$$

where Ds is Simpson's index of diversity, N is the total number of samples, and n_i_ is the number of samples in the i^th^ group.

## 3. Results

### 3.1. CRISPR analysis

**Figure 1.**
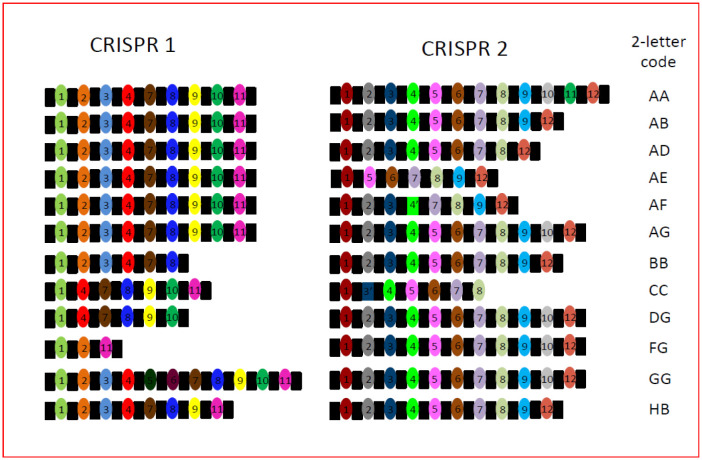
Graphical representation of the spacer patterns identified for the two CRISPR loci in this study and their corresponding two-letter binary codes. Black rectangles represent the direct repeat sequences, while color coded ovals represent the spacer sequences; the latter are also identified numerically according to their 5′ to 3′ orders. Two significant variations in the spacer sequences are identified by rectangles in place of ovals in the CRISPR 2 graphic; these are identified as 3′ and 4′, in place of 3 and 4, respectively. Spacers 9, 10 and 11 of CRISPR 2 were identical in sequence. The two-letter binary code is indicated to the right of each combined CRISPR profile.

All 92 strains examined generated both CRISPR 1 and CRISPR 2 loci for sequencing. The sequence of the 29 base DR was highly conserved within the CRISPR 1 locus, while that of the CRISPR 2 locus exhibited much more variation, with base substitutions observed in several samples (Table 4). The CRISPR 1 locus comprised up to 11 spacer sequences, while the CRISPR 2 locus comprised up to 12, with significant sequence variations in spacers 3 and 4 for individual strains such that they are identified as 3′ and 4′. Full details of all spacer sequences, including all variations thereof, are presented in Table S1, while Figure 1 summarises the combinations of spacers that defined each CRISPR subtype. A total of 7 spacer combinations, labelled with a single letter code (A to G), were observed for each CRISPR locus (Figure 1). Combining the letter codes for each CRISPR locus generated a two-letter binary code, with additional small variations due to base substitutions indicated by a numerical code (1, 2, etc.) (Table S1). When both CRISPR loci sequences were combined, they identified a total of 16 distinct *S*. Enteritidis subtypes. In addition, the P125109 reference strain yielded sequences identical to those published previously for the two CRISPR loci and was assigned to the HB subtype based on our classification scheme.

**Table 4.** Sequences of all direct repeat motifs identified in both CRISPR loci.

CRISPR		
1	Consensus repeat	CGGTTTATCCCCGCTGGCGCGGGGAACAC
	5′ terminal repeat	GTGTTTATCCCCGCTGACGCGGGGAACAC

CRISPR		
2	Consensus repeat	CGGTTTATCCCCGCTGGCGCGGGGAACAC
	5′ terminal repeat	ACGGCTATCCTTGTTGGCGCGGGGAACAC
	variation (single isolate)	ACGGCTATCCTGGTTGGCGCGGGGAACAC
	Internal repeat variants	GGGTTTATYCCCGCTGGCGCGGGGAACAA
		GGGTTTATCCCCGCTGGCGCGGGGAACAC
		CGGTTTATCCCCGCTGGCGAGGGGAACAC
		CGGTTTATCCCCGATGGCGCGGGGAACAC
		CGGTTTATCTCCGCTGGGGCGGGGAACAC
		CGGTCTATCCCCGCTGGCGCGGGGAACAC
		CGCTTTATCCCCGCTGGCGCGGGGAACAC
	Internal and 3′ terminal repeat	CAGTTTATCCCCGCTGGCGCGGGGAACAC

Bases different from those of each consensus sequence are underlined		

### 3.2. Comparison with other S. Enteritidis typing schemes

These 92 strains yielded 19 different PFGE patterns, with the majority falling into three groups: SENXAI.0003_ SENBNI.0003 (n = 35), SENXAI.0006_ SENBNI.0007 (n = 14) and SENXAI.0038_ SENBNI.0016 (n = 15). Six samples comprised the PFGE type SENXAI.0214_ SENBNI.0225, while the remaining 15 subtypes were distributed among 22 strains.

Of the 26 different phage types (PTs) identified amongst this collection, the most common were PT8 (n = 23), PT13 (n = 13), PT13a (n = 9) and PT23 (n = 13). Eleven atypical PTs were observed, two of which were unattributed to a group, and one sample could not be typed. Four strains appeared to be either of mixed type or to have modified their PT during assessment; for the purpose of Simpson's index of diversity calculation, as detailed below, the rarer type was employed.

**Figure 2.**
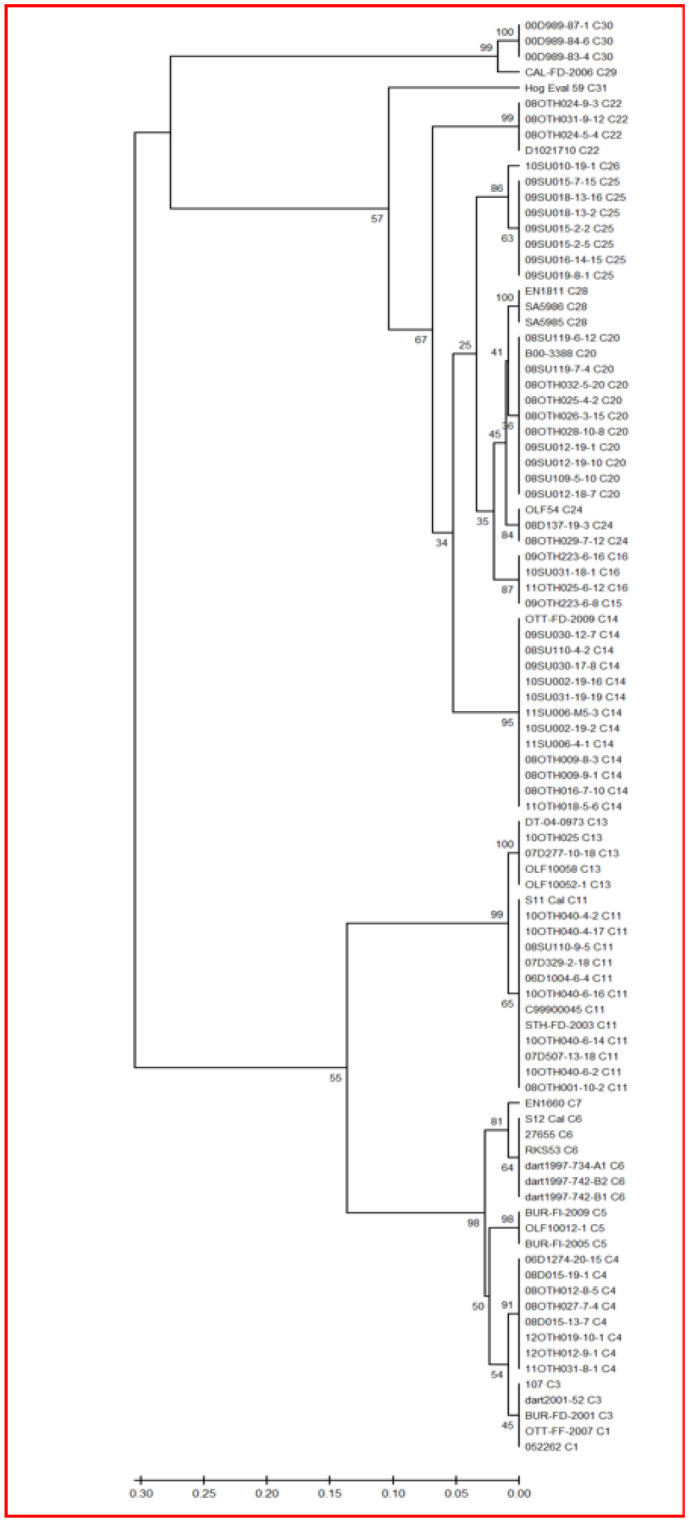
Phylogeny of the 92 *S*. Enteritidis isolates based on their SNP-PCR profiles. For each sample, a concatenated set of specific nucleotides at 60 genomic positions was compiled. The combined fasta file for all samples was used to generate a phylogenetic tree using the UPGMA method, with 1000 bootstrap replicates in MEGA X. The cladal subtype is indicated for each isolate after its sample designation and bootstrap values are indicated at all nodes. Separations of clades 1 and 3 and clades 15 and 16 were not achieved in this illustration due to the software's pairwise deletion of positions that include gaps; such gaps are the sole means of differentiating these subtypes.

SNP-PCR typing scored the base sequence of 60 positions scattered throughout the genome and subsequently used these concatenated data for phylogenetic tree generation. Based on hundreds of *S*. Enteritidis samples, this process has been reported to identify many distinct clades [25], of which 20 were identified in the sample collection (Figure 2).

Simpson's index of diversity was calculated for each subtyping method, as summarised in Table 5; the full calculation summary of these values is provided (Table S2). This clearly shows that, for the sample set examined in this study, the CRISPR analysis was the least discriminatory, identifying just 16 subtypes with an index of diversity of 0.7367. Although PT identified the most subtypes (n = 25), the SNP-PCR subtyping, which identified 20 subtypes, had an index of diversity of 0.9259 and was scored as the most discriminatory compared to the traditional methods of PFGE and PT.

**Table 5.**
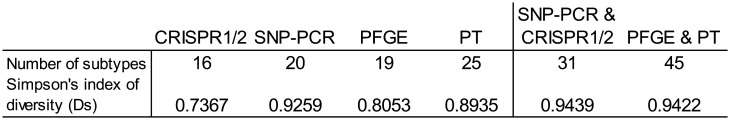
Metrics yielded by each subtyping method separately and in paired combinations.

**Figure 3.**
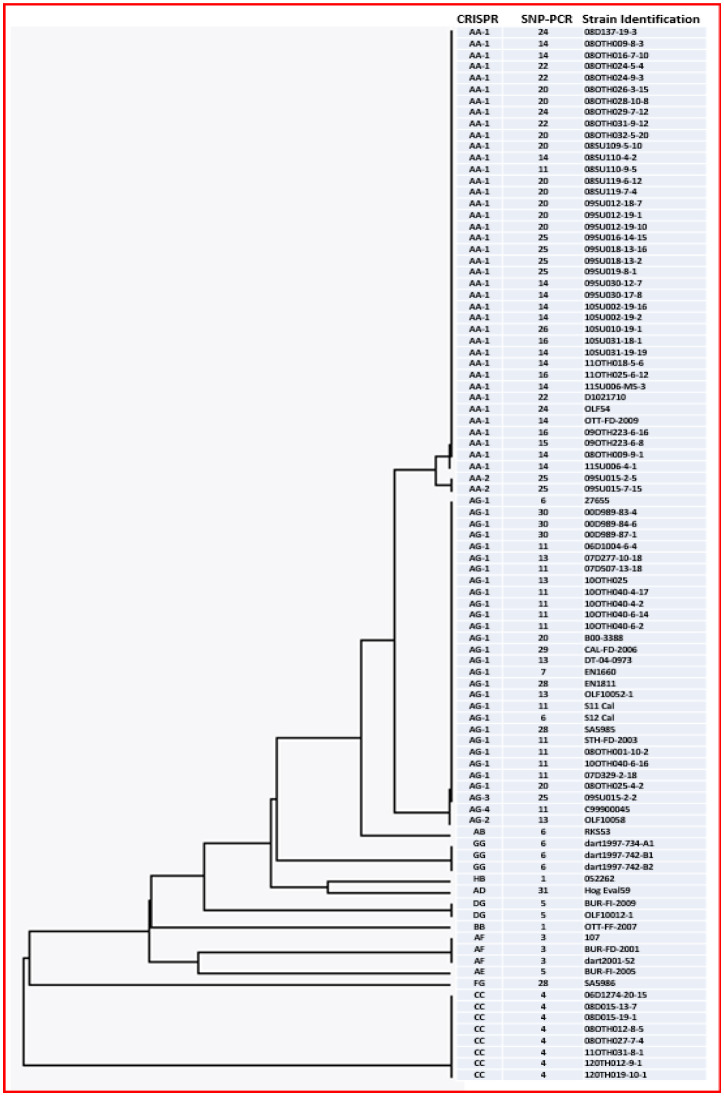
Phylogenetic tree of the 16 CRISPR subtypes identified in this cohort of 92 samples as generated using the UPGMA method. CRISPR and SNP-PCR subtypes as well as sample designations are shown.

When subtyping methods were paired, not surprisingly, their discriminatory capabilities increased; the combined discriminatory capabilities of the two traditional methods versus the two molecular methods are summarised (Table 5). Comparison of the subtyping assignments for individual samples did reveal some level of concordance between methods for some subtypes, as well as significant variations in subtype structure for many samples (Table 1 and Table S3). This is illustrated by the phylogenetic tree (Figure 3) representing the CRISPR subtypes for all samples, to which has been added the corresponding SNP-PCR data. For example, all eight strains with a CC CRISPR subtype were classified as SNP-PCR subtype 4, but most other CRISPR subtypes were scattered amongst several SNP-PCR subtypes. The two most dominant CRISPR subtypes, namely AA-1 (n = 39) and AG-1 (n = 26), were each classified into multiple separate SNP-PCR subtypes. The AA-1 CRISPR subtype included strains belonging to SNP-PCR subtypes 11, 14, 15, 16, 20, 22, 24, 25 and 26, whereas the AG-1 CRISPR subtype was composed of SNP-PCR subtypes 6, 7, 11, 13, 20, 28, 29 and 30. Conversely, some SNP-PCR subtypes are restricted to a specific CRISPR subtype (e.g., SNP-PCR subtypes 14, 15, 16, 22, 24 and 26 all correspond to CRISPR subtype AA-1), but this is not always the case. Often, each SNP-PCR subtype is composed of two or three CRISPR subtypes, e.g., SNP-PCR 5 (AE and DG CRISPR subtypes), SNP-PCR 6 (AB, AG-1 and GG CRISPR subtypes) and SNP-PCR 25 (AA-1, AA-2 and AG-3 CRISPR subtypes). As a result, when both typing methods were combined, the number of distinct subtypes increased to 31, with an index of diversity of 0.9439, which was the highest observed in this study. Combining PFGE and PT analysis yielded the greatest number of distinct subtypes at 45, yet the increased index of diversity of 0.9422 observed for the combined traditional methods was marginally lower than for the combined molecular methods of CRISPR/SNP-PCR subtyping.

### 3.3. Sequence analysis of the fimH gene

As the sequencing of additional genes in concert with CRISPR analysis has been reported to improve *S*. Enteritidis subtype discrimination [33], an initial screen of 70 strains employed in this study, including the P125109 strain used as the reference, was undertaken to explore the utility of including the sequence of the 1008 bp *fim*H gene in the subtyping scheme. Based upon the *fim*H gene sequence of the reference, which was scored as allele A, only three other alleles of this gene were identified based on SNPs at nine positions (Tables 1 and 3). However, seven of these SNPs were associated with the two distinctive strains with a DG CRISPR profile, one was found in the single strain with an AE CRISPR profile, and the last SNP was found in a single strain of CRISPR type AG-1. As a result, this additional sequence analysis added only a single subtype to this set of 70 samples.

## 4. Discussion

The primary application of bacterial typing in disease control is to reveal epidemiological links between isolates responsible for human illness and the source of the causative agent. For outbreaks of foodborne gastroenteritis, identifying the food product contaminated with a specific bacterial pathogen enables its recall and limitation of the disease outbreak. Such investigations require timely action on the part of public health officials, thereby limiting the extent of the outbreak; this in turn requires efficient laboratory identification of the strain of the bacterium involved. Given the importance of *S*. Enteritidis in human foodborne disease, this study has explored sequence typing of the two CRISPR loci as a rapid method of potential utility for such investigations.

However, CRISPR analysis of the 92 strains included in this study was less discriminatory compared to the traditional typing methods of PFGE and PT. While CRISPR typing did clearly distinguish strains recovered from distinct food sources such as seafood (clams), a high number of isolates fell into two CRISPR groups, AA-1 (n = 39) and AG-1 (n = 26), representing 42.4% and 28.3%, respectively, of all samples. With few exceptions, these isolates were all strongly associated with poultry production facilities or poultry food products, often with multiple samples recovered from single submissions. Given that these are the products primarily responsible for many human infections, this clearly limits the ability of CRISPR-based typing alone to locate the precise source of many food contaminants. Clearly the interpretation of the value of this approach will depend on the range of isolates requiring discrimination.

Comparison of the subtyping assignments by these different methods revealed very limited concordance in their grouping patterns, except for a set of eight samples that were clearly discriminated by their CRISPR CC subtype, a SENBNI.0225 PFGE profile, a PT of 9b or a closely related atypical subtype and SNP subtype 4. The close evolutionary relationship of these samples, which all originated from duck producing facilities and which harbour an unusual virulence plasmid, has been reported previously [46]. The lack of similar concordance in the subtyping profiles for most other samples reflects the independent nature of the features being targeted, and, while this does not limit their utility for strain identification, it suggests that in general many of these targets may not reveal the evolutionary paths of the strains tested. Nevertheless, the SNP-PCR method, when applied to a large population of *S*. Enteritidis (n = 1,227), provides insights into the genetic structure of the organism [25].

Some studies, in which CRISPR sequence analysis has been combined with that of two additional genes, *fimH* and *sseL* (CRISPR-MVLST), have reported satisfactory discriminatory capability [33],[34]. Indeed, given that the CRISPR-MVLST typing scheme for *S*. Enteritidis has been reported to be more discriminatory than PFGE [47], the value of including additional sequence information for these two genes was explored. However, neither target was found to significantly improve discriminatory capability for this sample cohort. The *fimH* locus identified just a single additional type out of 70 of the 92 isolates of this study, while review of whole genome sequence data previously reported for some of these isolates [48] indicated that the *sseL* gene was highly conserved. Given the additional effort involved in the amplification and sequencing of these targets, their inclusion in this subtyping scheme was not considered worthwhile.

Combination of CRISPR-MVLST with PFGE has been reported to improve discriminatory capability for clinical *S*. Enteritidis isolates [49]. Indeed, in this study the CRISPR/SNP-PCR combination, which identified 31 groups, yielded a marginally higher index of diversity than the PFGE/PT subtyping combination, even though the latter identified a much higher number of groups. However, the significant technical challenges posed by the PT method and concerns over the epidemiological significance of this subtyping tool, due to reports that some *Salmonella* serovars can change PT [50],[51], undermine its value for timely food borne illness investigations. Indeed, this problem was identified in four samples of this study for which a single distinct PT result was not obtained. This suggests a high number of groups, in itself, may have limited biological value, and other measures of sample diversity may be more meaningful.

## 5. Conclusion

This study provides a cautionary note on the use of CRISPR characterisation for *S*. Enteritidis subtyping. While CRISPR analysis alone did provide some discriminatory capability, in isolation this method was inferior to the other subtyping methods examined in this study. Even with the addition of *fim*H gene sequencing, it was not found to be a sufficiently sensitive subtyping tool. Indeed, this study reinforces prior observations that these loci are relatively conserved across this serovar and thus do not appear to reflect recent acquisition of spacer sequences [38], thereby diminishing their use for high resolution epidemiological studies. Combining SNP-PCR and CRISPR sequence determination, analyses more readily performed by the standard microbiology laboratory and in a more timely fashion than the traditional techniques, would offer an alternative to those laboratories lacking the necessary hardware or bioinformatics support for whole genome sequence analysis.
